# Supplementary material for: Antibiotic Use and Long-Term Outcome in Patients with Tick-Borne Encephalitis and Co-Infection with Borrelia Burgdorferi Sensu Lato in Central Europe. A Retrospective Cohort Study
Source: J Clin Med. 2019 Oct 20;8(10):1740. doi: 10.3390/jcm8101740 (PMC6832614; doi:10.3390/jcm8101740)
Supplement: Supplementary file 1 [file jcm-08-01740-s001.pdf]

Journal of
Clinical Medicine

Supplementary Materials

Table S1. Demographic, clinical, and laboratory characteristics of patients with tick-borne encephalitis on admission in relation to positive or negative test result for IgM or IgG antibodies to *B. burgdorferi* sensu lato.

Characteristic	***Borrelia* seropositive** ***n* = 282**	***Borrelia* seronegative** ***n* = 402**	***p* Value**
Age	59 (46–69)	49 (35–61)	<0.001
Male sex	176 (62.4)	219 (54.5)	0.047
Charlson comorbidity index	2 (0–3)	1 (0–2)	<0.001
Vaccinated against TBE	14 (5.0)	14 (3.5)	0.443
Clinical presentation			
Meningitis	61 (21.6)	96 (23.9)	
Meningoencephalitis	196 (69.5)	270 (67.2)	0.778
Meningoencephalomyelitis	25 (8.9)	36 (9.0)	
Severity of acute illness			
Mild	103 (25.7)	66 (23.4)	
Moderate	246 (61.4)	169 (59.9)	0.374
Severe	52 (13.0)	47 (16.7)	
Severity score of acute illness	12 (9–19)	12 (8–17)	0.229
CSF leukocyte count (x 10^6/L)	75.5 (40–138)	105 (55–188.3)	**<0.001**

Data are median (interquartile range) or number (%) of patients. Abbreviations: CSF, cerebrospinal fluid.

Table S2. Demographic, clinical, and laboratory characteristics of patients with tick-borne encephalitis and possible borrelial co-infection according to whether or not they received anti-borrelial antibiotic therapy.

**Characteristic**	**Anti-borrelial antibiotic therapy**		***p* Value**
	**Yes** ***n* = 144**	**No** ***n* = 96**	
**Age**	61 (50.8–69)	53.5 (41.8–67)	**0.006**
Male sex	94 (65.3)	54 (56.3)	0.203
Charlson comorbidity index	2 (1–3)	1 (0–3)	**0.006**
Vaccinated against TBE	9 (6.3)	3 (3.1)	0.371
Clinical presentation			
Meningitis	35 (24.3)	15 (15.6)	0.268
Meningoencephalitis	97 (67.4)	72 (75.0)	
Meningoencephalomyelitis	12 (8.3)	9 (9.4)	
Severity of acute illness			
Mild	35 (24.3)	19 (19.8)	0.554
Moderate	83 (57.6)	62 (64.6)	
Severe	26 (18.1)	15 (15.6)	
Severity score of acute illness	13 (9–20)	12 (10–18.3)	0.858
CSF leukocyte count (x 10^6/L)	67 (35–129.3)	74.5 (34.5–138.3)	0.968

Data are median (interquartile range) or number (%) of patients.

Table S3. Number (%) of patients with tick-borne encephalitis and possible borrelial co-infection who had incomplete recovery at follow-up visits in relation to whether or not they received anti-borrelial antibiotic therapy.

	**All**	**Anti-borrelial antibiotic therapy**		***p* Value** [a]
	***n* = 240**	**Yes** *n* **= 144**	**No** *n* **= 96**	
2 months post-enrolment	129/232 (55.6)	77/140 (55.0%)	52/92 (56.5%)	0.926
6 months post-enrolment	72/192 (37.5)	48/122 (39.3)	24/70 (34.3)	0.588
12 months post-enrolment	41/128 (32.0)	29/94 (30.9)	12/34 (35.3)	0.794
At final evaluable visit	75/233 (32.2)	43/140 (30.7)	32/93 (34.4)	0.654

[a] Overall p value for comparisons between groups was estimated using the normal approximation with continuity correction; $p < 0.05$ was considered significant.
